# Comparative Analysis of Laboratory Markers, Severity Scores, and Outcomes in 179 Patients with Severe Acute Pancreatitis

**DOI:** 10.3390/biomedicines13040797

**Published:** 2025-03-26

**Authors:** Tudorel Mihoc, Catalin Pirvu, Amadeus Dobrescu, Dan Brebu, Anca Monica Oprescu Macovei, Stelian Pantea, Claudia Borza, Patrick Dumitrescu, Monica Laura Cara

**Affiliations:** 1 Department X, Surgical Emergencies Clinic, “Victor Babeș” University of Medicine and Pharmacy Timișoara, 300041 Timișoara, Romania; mihoc.tudorel@umft.ro (T.M.); pirvu.catalin@umft.ro (C.P.); pantea.stelian@umft.ro (S.P.); 2 Department X, 2nd Surgical Clinic, Researching Future “Chirurgie 2”, “Victor Babeș” University of Medicine and Pharmacy Timișoara, 300041 Timișoara, Romania; dobrescu.amadeus@umft.ro (A.D.); dr.brebudan@gmail.com (D.B.); 3 Department of Gastroenterology, Emergency Hospital Prof. Dr. Agripa Ionescu, “Carol Davila” University of Medicine and Pharmacy, 050474 Bucuresti, Romania; anka_makovei@yahoo.com; 4 Department of Functional Sciences–Pathophysiology, “Victor Babeș” University of Medicine and Pharmacy Timișoara, 300041 Timișoara, Romania; 5 Centre for Translational Research and Systems Medicine, “Victor Babeș” University of Medicine and Pharmacy Timișoara, 300041 Timișoara, Romania; 6 Faculty of Medicine, “Victor Babeș” University of Medicine and Pharmacy Timișoara, 300041 Timișoara, Romania; patrick.dumitrescu@student.umft.ro; 7 Department of Public Health and Management, University of Medicine and Pharmacy of Craiova, 200349 Craiova, Romania; monica.cara@umfcv.ro

**Keywords:** acute pancreatitis, inflammatory markers, severity scores, mortality, organ failure

## Abstract

**Background and Objectives**: Severe acute pancreatitis carries a substantial risk of complications and death. Prompt identification of prognostic factors is crucial to optimize management and reduce mortality. This study aims to compare inflammatory scores, laboratory markers, and clinical outcomes between survivors and non-survivors with severe acute pancreatitis, drawing on data from 179 patients admitted between 2017 and 2024. **Methods**: We conducted a retrospective cohort study of 179 patients diagnosed with severe acute pancreatitis. Of these, 55 patient records were extracted from an existing database, and an additional 124 were included from hospital archives (2017–2024). We divided participants into survivors (*n* = 121) and non-survivors (*n* = 58). Clinical data were obtained from medical records, including demographic information, comorbidities, laboratory markers (neutrophil-to-lymphocyte ratio (NLR) and platelet-to-lymphocyte ratio (PLR)), and severity scores (Acute Physiology and Chronic Health Evaluation (APACHE), Computed Tomography Severity Index (CTSI), and Ranson). **Results**: Non-survivors had significantly higher ages (mean of 66.4 vs. 52.7 years, *p* = 0.002), elevated inflammatory markers (median NLR of 14.2 vs. 10.3, *p* = 0.031), and more frequent multiorgan failure (75.9% vs. 31.4%, *p* < 0.001). The timing of intervention before 28 days was associated with higher mortality (*p* = 0.004). Chronic kidney disease and advanced cardiovascular comorbidities independently predicted worse survival (*p* = 0.009). The mortality rate in this cohort was 32.4%. Logistic regression identified age >60 years with an odds ratio (OR = 2.9), multiple organ failure (OR = 4.1), and high severity scores as primary contributors to mortality. **Conclusions**: Advanced age, comorbidities, elevated inflammatory markers, and multiple organ failure significantly impact mortality in severe acute pancreatitis. Delaying major interventions when feasible, optimizing perioperative care, and early recognition of high-risk patients may improve outcomes. Further research should explore targeted management strategies for high-risk groups and refine the role of delayed or minimally invasive approaches in severe acute pancreatitis management.

## 1. Introduction

Severe acute pancreatitis (SAP) is an aggressive inflammatory disorder of the pancreas that can lead to extensive local and systemic complications [1]. Although acute pancreatitis is often self-limiting, approximately 15–20% of patients develop a severe form associated with organ failure and high mortality [2]. The transition from mild or moderate to severe disease involves complex immune-mediated pathways and microcirculatory disturbances, culminating in pancreatic necrosis, systemic inflammatory response syndrome (SIRS), and, in some cases, multiple organ dysfunction [3].

Despite advances in intensive care, mortality in SAP remains significant, ranging between 15% and 30% in reported series [4]. The revised Atlanta classification highlights persistent organ failure as a central determinant of SAP, emphasizing early risk stratification [5]. Moreover, the extent of pancreatic and peripancreatic necrosis strongly correlates with the development of infected necrosis, which exacerbates clinical deterioration [6]. Early identification of at-risk patients is therefore paramount to optimize therapeutic interventions and improve outcomes [7].

Several scoring systems have been developed to facilitate early recognition of severe disease and stratify the need for intensive care. The Acute Physiology and Chronic Health Evaluation (APACHE) II score offers a broad physiologic assessment and is commonly used in critical care [8]. Ranson’s criteria remain historically significant, although they require data over a 48 h period [9]. Morphologically, contrast-enhanced computed tomography (CECT) findings are quantified by the Computed Tomography Severity Index (CTSI), which incorporates both the extent of pancreatic inflammation and necrosis [10]. Integrating these tools with bedside assessments provides a more holistic approach to risk classification [6].

In parallel, various laboratory markers have been proposed to improve prognostic accuracy. C-reactive protein (CRP) is a well-established acute-phase reactant that tends to peak 48–72 h after the onset of pancreatitis and correlates with severity [11]. More recently, the neutrophil-to-lymphocyte ratio (NLR) and platelet-to-lymphocyte ratio (PLR) have gained attention as inexpensive and readily available predictors of adverse outcomes [12]. Elevated values in these indices reflect systemic inflammation and immune dysregulation, which frequently precede organ dysfunction [13].

Management of SAP has shifted from early aggressive surgical debridement to a more conservative or step-up approach. Minimally invasive drainage techniques and endoscopic necrosectomy have demonstrated efficacy in reducing morbidity and mortality in selected cases [14]. Nonetheless, open necrosectomy remains essential for patients who fail to respond to less invasive measures or who present with extensive necrosis and hemodynamic instability [15]. Determining the optimal timing of any intervention—particularly if necrosis becomes infected—remains a critical challenge [16].

Research continues to explore comprehensive strategies for reducing mortality in SAP, including improved supportive care in the intensive care setting and closer monitoring of biochemical markers [17]. Pre-existing comorbidities such as cardiovascular disease, diabetes mellitus, and especially chronic kidney disease appear to exacerbate the course of pancreatitis [18,19]. Therefore, this study aims to enhance the understanding of severe acute pancreatitis by comparing inflammatory scores, laboratory markers, and clinical outcomes between survivors and non-survivors, offering a unique approach by integrating a comprehensive array of prognostic factors collected over a significant seven-year span. The novelty of our research lies in its rigorous examination of the timing of surgical interventions in relation to patient outcomes, a less-explored aspect in the existing literature. By focusing on detailed clinical data, including demographic information, comorbidities, and a range of severity scores such as APACHE, the CTSI, and Ranson, this study aims to substantiate the benefits of delayed interventions and provide a refined strategy for managing high-risk patients, potentially setting a new benchmark for clinical practices in severe acute pancreatitis management.

## 2. Materials and Methods

### 2.1. Study Design and Population

A retrospective cohort study was conducted at a tertiary center in Timisoara, Romania from January 2017 to December 2024. Patients older than 18 years with a confirmed diagnosis of severe acute pancreatitis (based on the revised Atlanta classification) were screened. Inclusion criteria required persistent organ failure for over 48 h, while exclusion criteria encompassed incomplete data, transfer from another institution after major interventions, or alternative causes of pancreatitis unrelated to acute inflammation. A total of 179 patients met eligibility requirements, comprising 121 survivors and 58 non-survivors.

Patients’ demographic information (age, sex, and comorbidities) and etiological factors (biliary, alcoholic, hypertriglyceridemia, or other) were recorded. The primary outcome was mortality, and secondary outcomes included length of hospital stay, need for intensive care unit (ICU) admission, and complications (infected necrosis and organ failure). Ethical approval was obtained from the hospital review board, and patient anonymity was preserved throughout data collection.

### 2.2. Clinical and Laboratory Data

On admission, patients underwent a standardized assessment that included vital signs, complete blood count, and biochemical tests (serum amylase, lipase, creatinine, blood urea nitrogen, and CRP). The NLR and PLR were calculated. Organ failure was classified based on Marshall score criteria, identifying respiratory, renal, or circulatory dysfunction.

Severity scoring systems included the APACHE II, Ranson’s criteria (evaluated at admission and after 48 h), and the CTSI [20]. CT scans were performed per clinical necessity. Patients’ laboratory profiles and clinical features were documented in an electronic database, ensuring consistent variable coding.

### 2.3. Management Protocol

All patients initially received aggressive fluid resuscitation, pain control, and nutritional support (preferably early enteral feeding). Antibiotics were administered only when infected necrosis or another source of infection was confirmed. Indications for invasive intervention followed a stepwise approach: percutaneous or endoscopic drainage was attempted when infected collections were identified unless contraindicated by patients’ hemodynamic instability or extensive necrosis requiring open surgical intervention.

For those requiring necrosectomy, either laparoscopic or open approaches were chosen based on the location and extent of necrosis, surgical expertise, and the patient’s overall status. The timing of surgical debridement was categorized as early (<28 days from onset) or delayed (≥28 days). All decisions were made through multidisciplinary consensus involving surgeons, gastroenterologists, radiologists, and ICU physicians.

### 2.4. Data Analysis

An a priori sample size calculation was conducted using the estimated mortality rate of approximately 30% in severe acute pancreatitis and an anticipated 15% difference between groups. We set the power at 80% and alpha at 0.05, leading to the requirement of at least 165 patients. Ultimately, 179 individuals were enrolled to accommodate potential exclusions and strengthen this study’s statistical reliability. Data were extracted from paper and electronic records and entered into a spreadsheet for validation and statistical evaluation. Continuous variables were examined for normality and presented as means with standard deviations or medians with interquartile ranges, as appropriate. Group comparisons (survivors vs. non-survivors) were performed using the independent *t*-test or Mann–Whitney U test. Categorical data were summarized as counts and percentages, with the Chi-square or Fisher’s exact test employed for comparisons. Logistic regression was used to identify independent predictors of mortality, incorporating significant univariate variables. Odds ratios (ORs) and 95% confidence intervals were reported. No sample size calculation was performed a priori given the retrospective design, but the final dataset of 179 patients was deemed sufficient to detect key differences in mortality predictors. Statistical significance was defined as *p* < 0.05 for all tests.

## 3. Results

Data from 179 patients with severe acute pancreatitis are presented in Table 1. Non-survivors were older on average (66.4 vs. 52.7 years), with the difference reaching statistical significance (*p* = 0.002). The proportions of different etiologies (biliary, alcohol, hypertriglyceridemia, or other) did not differ substantially between the two groups; biliary pancreatitis remained the most frequent cause overall. Although multiple comorbidities were more prevalent in non-survivors (50.0%) than survivors (36.4%), the *p*-value of 0.076 suggests a trend rather than a definitive significance in this sample size. Importantly, 100% of non-survivors required ICU admission, indicating a higher initial severity or rapid clinical deterioration compared to survivors (78.5% ICU admission). Early necrosectomy (within 28 days from symptom onset) occurred in nearly half (44.8%) of the non-survivors, while a smaller proportion of survivors (24.0%) underwent early surgery.

White blood cell counts were significantly higher in non-survivors (16.1 vs. 13.2 × 10^9^/L, *p* = 0.001), reflecting an enhanced inflammatory or infectious process. Similarly, the median NLR was significantly increased in non-survivors (14.2) compared to survivors (10.3), suggesting that a heightened systemic inflammatory response correlates with a worse prognosis (*p* = 0.031). The PLR also showed a noticeable elevation in non-survivors (218.6 vs. 195.2, *p* = 0.012). CRP displayed a marked difference: mean values in non-survivors approached 249.8 mg/L, significantly higher than the observed value of 187.6 mg/L in survivors (*p* < 0.001), as seen in Table 2.

**Figure 1:**
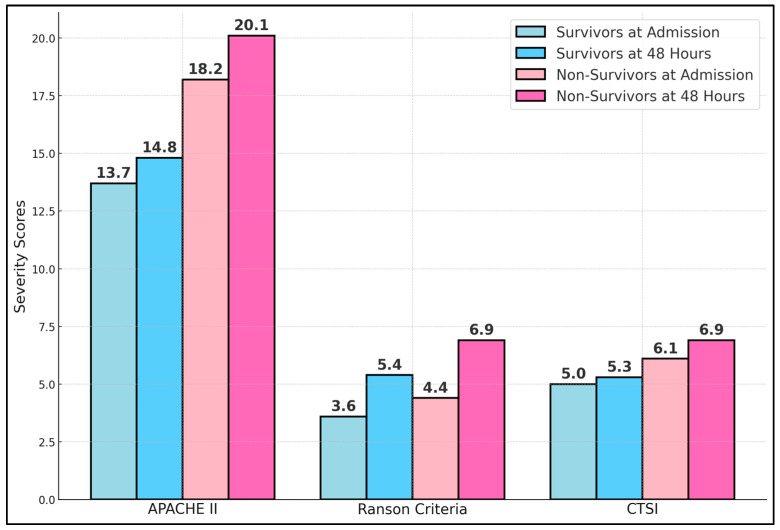
Comparison of severity scores at admission and 48 h.

Table 3 compares common severity scoring systems—APACHE II, Ranson, and the CTSI—in survivors versus non-survivors at two critical time points: hospital admission and 48 h later. Non-survivors presented significantly higher APACHE II scores both at admission (18.2 vs. 13.7, *p* < 0.001) and at 48 h (20.1 vs. 14.8, *p* < 0.001). This aligns with previous findings that APACHE II effectively stratifies severity in acute pancreatitis, especially when measured dynamically. Ranson’s criteria also revealed meaningful differences, with non-survivors averaging 4.4 points at admission compared to 3.6 among survivors (*p* = 0.008). By 48 h, the gap widened further (6.9 vs. 5.4, *p* < 0.001), reflecting the progression of disease and organ failure. The CTSI, which accounts for morphological changes in the pancreas and peripancreatic tissues, demonstrated that non-survivors had consistently higher scores (6.1 vs. 5.0 at admission; 6.9 vs. 5.3 at 48 h), as presented in Figure 1.

A significant finding is that only 8.6% of non-survivors were managed conservatively without drainage or surgery, in stark contrast to 31.4% of survivors (*p* < 0.001). This suggests that patients requiring no invasive interventions may be inherently less severe or have more favorable early responses to medical management. Endoscopic or percutaneous drainage was more common among survivors (36.4% vs. 17.2%, *p* = 0.012), potentially indicating that less invasive “step-up” strategies were effective in stabilizing those patients. Meanwhile, necrosectomy was more common in non-survivors (74.1% vs. 32.2%, *p* < 0.001). The timing of surgical intervention is also informative: 37.9% of non-survivors underwent surgery before 28 days, which is nearly triple that of the rate in survivors (11.6%, *p* < 0.001). Early surgical intervention is often precipitated by rapid clinical deterioration or uncontrolled sepsis but may increase operative complexity. Even surgery after 28 days, while potentially less hazardous, was still more frequent in non-survivors (36.2% vs. 20.7%, *p* = 0.038). Finally, the mean operative duration was approximately 15 min longer in non-survivors who underwent necrosectomy, with a statistically significant difference (*p* = 0.016), as presented in Table 4.

Among the 179 patients included in this study, 82 (45.8%) ultimately required necrosectomies. Of these, 50 (60.9%) underwent an open surgical approach via a laparotomy, while 32 (39.1%) were managed with a laparoscopic “step-up” technique. The overall in-hospital mortality rate for patients undergoing open necrosectomy was 44.0%, compared with 25.0% for those who received laparoscopic necrosectomy (*p* = 0.018). In contrast, patients who did not require surgery at all (*n* = 97, 54.2%) had a mortality rate of only 10.3%, likely reflecting their generally lower disease severity and success with conservative measures or percutaneous/endoscopic drainage. These findings suggest that while open necrosectomy may still be necessary in certain complex cases (extensive necrosis or hemodynamic instability), a laparoscopic approach—or avoiding surgery entirely—could confer better survival outcomes, highlighting the importance of individualized management strategies in severe acute pancreatitis.

Infected necrosis was significantly more prevalent in non-survivors (84.5%) than in survivors (58.7%, *p* = 0.001), consistent with the well-documented association between infected necrosis and higher mortality. Hemorrhagic complications requiring transfusion also showed a significant disparity (24.1% vs. 5.0%, *p* = 0.001), underlining the severity and fragility of non-survivors’ clinical states. Intestinal fistulas, although less common overall, emerged in 17.2% of non-survivors, compared to only 2.5% of survivors (*p* < 0.001). This suggests that more extensive necrosis and tissue friability, or perhaps repeated interventions, predispose to fistula formation. Another critical finding was the significantly higher rate of persistent organ failure post-operatively in non-survivors (62.1% vs. 9.9%, *p* < 0.001). Re-intervention rates further emphasize the complicated disease course in non-survivors (44.8% vs. 17.4%, *p* < 0.001), as described in Table 5.

Table 6 displays a logistic regression model evaluating independent predictors of mortality in severe acute pancreatitis. The strongest association emerges for multiple organ failure, with an odds ratio of 4.1 (95% CI, 2.2–7.9, *p* < 0.001), confirming the pivotal role of multiorgan dysfunction in driving fatal outcomes. An age of over 60 years also significantly elevates risk (OR = 2.9, *p* = 0.001), aligning with the clinical observation that older patients struggle more with the metabolic and inflammatory burden of severe pancreatitis.

**Figure 2:**
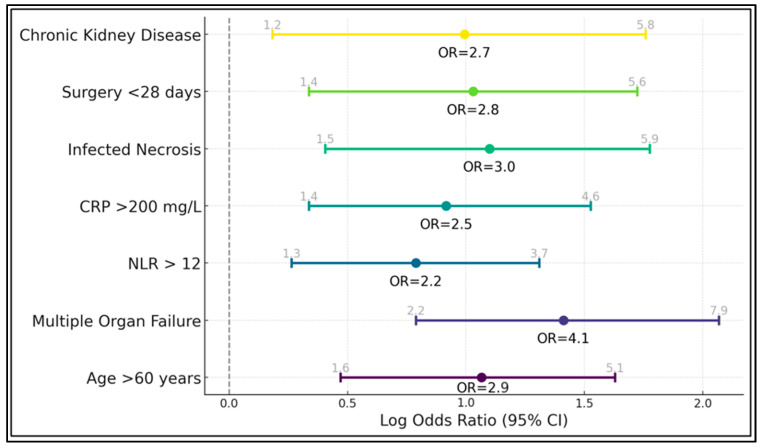
Risk factor analysis for mortality in severe acute pancreatitis.

Elevated inflammatory markers, particularly the NLR > 12 (OR = 2.2, *p* = 0.004) and CRP > 200 mg/L (OR = 2.5, *p* = 0.003), each independently predict mortality, indicating that an exaggerated immune response portends a worse trajectory. Infected necrosis, a grave complication, triples mortality risk (OR = 3.0, *p* = 0.002). Meanwhile, surgical intervention before 28 days doubles and nearly triples the odds of death (OR = 2.8, *p* = 0.003), echoing previous findings that early necrosectomy is often required by patients who are already severely deteriorated. Chronic kidney disease also significantly raises the likelihood of mortality (OR = 2.7, *p* = 0.014), as presented in Figure 2.

## 4. Discussion

Our findings underscore the importance of the early identification of high-risk patients with severe acute pancreatitis. Elevated inflammatory markers (NLR, PLR, and CRP) and persistent organ dysfunction emerged as key predictors of mortality. These results support the concept that both systemic inflammation and insufficient end-organ perfusion act synergistically to worsen outcomes. The association between an age of over 60 and increased mortality highlights the frailty of older patients, who often harbor comorbidities that compromise physiologic reserves. Notably, non-survivors frequently underwent surgical intervention earlier, likely indicating rapidly progressing necrosis or infection. Although this early operative approach is sometimes unavoidable, our data confirm that it correlates with worse prognoses.

The severity scoring systems (APACHE, Ranson, and the CTSI) demonstrated consistent discrimination between survivors and non-survivors, both on admission and at 48 h. Dynamic changes in these scores reflect the trajectory of the disease. By capturing evolving organ dysfunction, clinicians can detect early indicators of clinical deterioration and escalate care promptly. In line with emerging evidence, our analysis also emphasizes the role of minimally invasive techniques in selected patients, suggesting that endoscopic or percutaneous drainage might confer better outcomes if timed appropriately. However, open necrosectomy remains a lifesaving measure in refractory cases, particularly when infected necrosis threatens survival.

We propose the Pancreatitis Intervention Timing (PIT) Score to guide the timing of surgical intervention in severe acute pancreatitis. To calculate the score, assign 1 point for each of the following risk factors present: (1) age >60 years, (2) multiple organ failure, (3) neutrophil-to-lymphocyte ratio >12, (4) C-reactive protein >200 mg/L, (5) infected necrosis, (6) chronic kidney disease, and (7) advanced cardiovascular disease. Add the points to obtain a total PIT Score ranging from 0 (lowest risk) to 7 (highest risk). A higher PIT Score indicates an elevated risk of mortality and suggests that early, extensive surgical intervention (<28 days) should be approached with caution; for instance, a PIT Score of 4 or more may warrant delayed or minimally invasive procedures when feasible. Conversely, lower PIT Scores may allow for earlier intervention if clinically indicated. While the PIT Score offers a systematic framework for decision-making, it has not yet been prospectively validated and should therefore be interpreted as an adjunct to, rather than a replacement for, individualized clinical judgment.

Taken together, these results add to the accumulating body of literature advocating a tailored approach: “delay if possible, drain if indicated, debride if necessary”. Avoiding premature or extensive surgery may reduce complications, yet it is equally vital to intervene when conservative management fails or complications such as infected necrosis develop. By acknowledging the interplay of age, organ dysfunction, comorbidities, and inflammatory markers, clinicians can better prioritize ICU resources, adopt early supportive therapies (e.g., renal replacement and mechanical ventilation), and decide on an intervention strategy. Future prospective studies should investigate whether applying refined risk-based algorithms could further improve survival rates, optimize the timing of interventions, and reduce iatrogenic complications.

In recent studies on severe forms of acute pancreatitis (AP), significant findings have highlighted both the progression and outcomes of the disease. The systematic review by Sarri et al. [21] explored the burden of illness associated with moderately severe and severe acute pancreatitis (MSAP and SAP) across the USA and European Union-5, revealing that 15–20% of AP patients progress to these severe stages. Notably, the study found that up to 40% of those with SAP succumb to the disease during hospitalization, and up to 10.5% require surgical intervention for complications. In contrast, the prospective registry cohort study by Barrera Gutierrez et al. [22] focused on developing a prognostic model to predict SAP severity early in the treatment process. This study analyzed 516 patients and established baseline laboratory cutoff values for creatinine, white blood cells, procalcitonin, and systemic inflammatory response, which could predict SAP with a probability of 72% when all thresholds were exceeded. Both studies emphasize the critical need for early identification and intervention to improve outcomes in SAP, underscoring a substantial mortality rate and the potential for prediction-based management strategies to mitigate the progression and complications of the disease.

In the study by Yasuda et al. [23], the long-term outcomes of severe acute pancreatitis were analyzed in terms of recurrence, transition to chronic pancreatitis (CP), and the development of diabetes mellitus (DM). It was found that 19% of patients experienced a recurrence of AP, particularly those with necrotizing pancreatitis, and those patients exhibited higher levels of C-reactive protein and white blood cell counts. Transition to CP occurred in 22% of the patients and was notably more common among those with alcoholic SAP compared to biliary SAP, indicating a substantial impact of alcohol on chronic progression. Additionally, 39% of patients developed DM, with elevated blood glucose and base excess levels being predictive markers. In a similar manner, the systematic review by Giorga et al. [24] assessed long-term quality of life after SAP, revealing that while the quality of life was generally reduced, particularly in the first four years and more so in patients with alcoholic etiology, some could normalize over time. This study included 779 patients across 14 retrospective cohort studies, noting a variable quality of life impact, which was not universally detrimental. These findings suggest that while severe acute pancreatitis can lead to significant long-term health issues such as CP and DM, the impact on quality of life can vary greatly, with some patients recovering well, possibly due to advances in treatment modalities.

In the study by Füsun Adam et al. [25], the effectiveness of various scoring systems in predicting mortality in patients with severe acute pancreatitis admitted to the ICU was evaluated. The research demonstrated that the Sequential Organ Failure Assessment (SOFA) score was particularly effective, with an ICU mortality rate of 64% and a hospital mortality of 71%. Notably, patients with a SOFA score ≥11 at any point during their ICU stay exhibited a significantly higher mortality rate, indicating a robust predictive value with an area under the receiver operating characteristic curve (ROC) of 0.837. Conversely, the APACHE II score, while commonly used, did not show a significant correlation with mortality in this study. In a similar manner, the study by Jian-Hui Chen et al. [26] also utilized the APACHE II scoring system, but in the context of identifying risk factors for SAP complicated with acute gastrointestinal injury (AGI). This study identified an APACHE II score >15 and a creatinine level >100 µmol/L as significant independent risk factors for developing AGI in SAP patients, with a positive interaction between these factors indicating an increased risk when both are present. Both studies underscore the utility of specific scoring systems in predicting outcomes in severe acute pancreatitis, with SOFA proving superior in mortality prediction and APACHE II being effective in risk stratification for complications such as AGI.

The current study, being observational in nature, does not establish causality but rather identifies associations, such as the observed correlation between delayed surgery and improved survival rates in severe acute pancreatitis. It is crucial to acknowledge that patients who underwent surgery later may have had less severe initial presentations. Therefore, these conclusions are carefully framed to suggest that while delaying surgery appears beneficial, these findings are based on associative analysis and require further investigation through controlled clinical trials to confirm causative effects.

This study has several limitations. First, it is retrospective and thus subject to selection bias; clinicians’ decisions about the timing and type of intervention may have varied due to evolving protocols or resource availability. Second, while we combined data from 2017 to 2024, changes in diagnostic criteria or surgical techniques over time could have influenced outcomes. Moreover, not all radiological studies could be evaluated by the same expert radiologist, which could lead to interobserver variability in interpreting the CTSI. Lastly, the relatively small number of certain subgroups (e.g., hypertriglyceridemia pancreatitis) may limit the generalizability of etiologic findings. Ultimately, due to the retrospective nature of this study, patients were not followed after discharge to observe long-term complications and outcomes. Despite these constraints, this study offers valuable insights into predictors of mortality in severe acute pancreatitis and highlights key targets for clinical improvement.

## 5. Conclusions

In this comparative analysis of 179 patients with severe acute pancreatitis, we observed that age above 60, elevated inflammatory markers (NLR, PLR, and CRP), higher severity scores (APACHE, Ranson, and the CTSI), and the presence of persistent organ failure strongly predict mortality. Surgical timing emerged as a critical factor, with early necrosectomy (before 28 days) associated with worse outcomes, likely reflecting the severity of the underlying pathology in these patients. Chronic kidney disease further heightened mortality risk, emphasizing the role of comorbidities in modulating disease trajectory.

By delineating these risk factors, our findings suggest that treatment should be tailored to each patient’s dynamic clinical status. Minimally invasive interventions appear beneficial in stabilizing less critically ill individuals, whereas open necrosectomy, though essential in refractory or advanced cases, confers a higher complication rate. Optimal resource allocation, such as prioritizing patients with severe organ dysfunction for advanced ICU care, is essential. Monitoring standardized severity scores at admission and during the early course of hospitalization can facilitate timely escalation to invasive management or transfer to specialized centers. Ultimately, a proactive, multidisciplinary approach—combining critical care, endoscopic, surgical, and radiological expertise—remains paramount to improving survival and minimizing complications in severe acute pancreatitis.

## Figures and Tables

**Table 1:** Baseline demographic and clinical characteristics.

Variable	Survivors (*n* = 121)	Non-Survivors (*n* = 58)	*p*-Value
Age (years, mean ± SD)	52.7 ± 13.4	66.4 ± 11.2	0.002
Male Sex (%)	80 (66.1)	36 (62.1)	0.573
Biliary Etiology (%)	46 (38.0)	20 (34.5)	0.658
Alcoholic Etiology (%)	37 (30.6)	14 (24.1)	0.369
Hypertriglyceridemia (%)	10 (8.3)	5 (8.6)	0.945
Other Etiologies (%)	28 (23.1)	19 (32.8)	0.153
Multiple Comorbidities (%)	44 (36.4)	29 (50.0)	0.076
Mean Hospital Stay (days)	26.8 ± 8.9	24.2 ± 9.3	0.086
ICU Admission (%)	95 (78.5)	58 (100)	<0.001
Early Necrosectomy < 28 days (%)	29 (24.0)	26 (44.8)	0.004

**Table 2:** Laboratory markers upon admission.

Parameter	Survivors (*n* = 121)	Non-Survivors (*n* = 58)	*p*-Value
WBC (×10^9^/L)	13.2 ± 4.1	16.1 ± 5.2	0.001
NLR	10.3 (8.1–12.9)	14.2 (10.1–18.3)	0.031
PLR	195.2 ± 52.7	218.6 ± 55.8	0.012
CRP (mg/L)	187.6 ± 67.1	249.8 ± 80.4	<0.001
Serum Creatinine (µmol/L)	98.3 ± 23.7	134.2 ± 32.6	<0.001
BUN (mmol/L)	7.4 ± 2.3	10.1 ± 3.1	0.006
Serum Amylase (U/L)	624.9 ± 152.4	591.1 ± 162.8	0.224

**Table 3:** Severity scores at admission and 48 h.

Severity Score	Time Point	Survivors (*n* = 121)	Non-Survivors (*n* = 58)	*p*-Value
APACHE II	Admission	13.7 ± 3.1	18.2 ± 3.5	<0.001
	48 h	14.8 ± 3.2	20.1 ± 3.9	<0.001
Ranson Criteria	Admission	3.6 ± 1.3	4.4 ± 1.4	0.008
	48 h	5.4 ± 1.5	6.9 ± 1.7	<0.001
CTSI	Admission	5.0 ± 1.6	6.1 ± 1.5	0.002
	48 h	5.3 ± 1.7	6.9 ± 1.6	<0.001

**Table 4:** Interventional strategies and timing.

Intervention	Survivors (*n* = 121)	Non-Survivors (*n* = 58)	*p*-Value
Conservative Management Only (%)	38 (31.4)	5 (8.6)	<0.001
Endoscopic/Percutaneous Drainage (%)	44 (36.4)	10 (17.2)	0.012
Necrosectomy (%)	39 (32.2)	43 (74.1)	<0.001
Surgery <28 days (%)	14 (11.6)	22 (37.9)	<0.001
Surgery ≥28 days (%)	25 (20.7)	21 (36.2)	0.038
Mean Duration of Procedure (min)	140.6 ± 31.2	155.8 ± 30.1	0.016

**Table 5:** Post-operative complications.

Complication	Survivors (*n* = 121)	Non-Survivors (*n* = 58)	*p*-Value
Infected Necrosis (%)	71 (58.7)	49 (84.5)	0.001
Bleeding Requiring Transfusion (%)	6 (5.0)	14 (24.1)	0.001
Intestinal Fistula (%)	3 (2.5)	10 (17.2)	<0.001
Persistent OF Post-op (%)	12 (9.9)	36 (62.1)	<0.001
Re-intervention (%)	21 (17.4)	26 (44.8)	<0.001
90-day Mortality (%)	0 (0)	58 (100)	N/A

**Table 6:** Logistic regression of mortality risk factors.

Variable	Odds Ratio (95% CI)	*p*-Value
Age > 60 years	2.9 (1.6–5.1)	0.001
Multiple Organ Failure	4.1 (2.2–7.9)	<0.001
NLR > 12	2.2 (1.3–3.7)	0.004
CRP > 200 mg/L	2.5 (1.4–4.6)	0.003
Infected Necrosis	3.0 (1.5–5.9)	0.002
Surgery < 28 days	2.8 (1.4–5.6)	0.003
Chronic Kidney Disease	2.7 (1.2–5.8)	0.014

## Data Availability

Data availability is subject to hospital approval.
